# Smoking cessation Through Optimisation of clinical care in Pregnancy: the STOP randomised controlled trial

**DOI:** 10.1186/s13063-019-3653-4

**Published:** 2019-09-03

**Authors:** Brendan P. McDonnell, Patrick Dicker, Sheila Keogan, Luke Clancy, Carmen Regan

**Affiliations:** 1 Coombe Women & Infants University Hospital, Cork Street, Dublin 8, Ireland; 2 Royal College of Surgeons in Ireland, 123 St Stephen’s Green, Dublin 2, Ireland; 3 TobaccoFree Research Institute, Focas Research Institute, DIT Kevin Street, Camden Row, Dublin 2, Ireland

**Keywords:** Antenatal care, Smoking, Fetal growth restriction, Smoking cessation

## Abstract

**Background:**

Cigarette smoking negatively impacts on maternal and fetal health. Smoking cessation is one of the few interventions capable of improving pregnancy outcomes. Despite the risks, the most effective antenatal model of care for smokers is still unclear, and specific recommendations for screening for fetal growth restriction are absent.

**Methods:**

This is a pragmatic randomised controlled trial of a dedicated smoking cessation clinic versus routine antenatal care as an intervention to reduce cigarette smoking behaviour. Smoking mothers randomised to the Smoking cessation Through Optimisation of clinical care in Pregnancy (STOP) clinic will have all antenatal care provided by a team comprising an obstetrician, a midwife, and a smoking cessation practitioner. This intervention includes ultrasound screening for fetal growth restriction. The control arm comprises two groups: one receiving standard care with ultrasound screening for fetal growth restriction, and one receiving standard care with ultrasound screening for growth restriction only if clinically indicated by their healthcare provider. Four hundred and fifty women will be recruited and randomised to either intervention or control arms stratifying for age, parity, and history of fetal growth restriction.

**Results:**

The primary outcome is self-reported, continuous abstinence from smoking between the quit date and end of pregnancy, validated by exhaled carbon monoxide or urinary cotinine. The quit date is targeted as being at or before 16 weeks’ gestation and no further than 28 weeks’ gestation. The secondary outcomes are a set of variables including maternal and fetal morbidity and mortality, neonatal complications and delivery outcomes, smoking and psychological outcomes, and qualitative measures.

**Conclusions:**

Despite much research into cigarette smoking in pregnancy, the optimal model of care for these women is still unknown. This study has the potential to improve the model of antenatal care provided to pregnant women who smoke and to improve outcomes for both mother and infant.

**Trial registration:**

ISRCTN11214785. Registered on 8 February 2018.

**Electronic supplementary material:**

The online version of this article (10.1186/s13063-019-3653-4) contains supplementary material, which is available to authorized users.

## Background

Smoking in pregnancy is a risk factor associated with poor maternal and fetal outcome. It remains a significant cause of morbidity and mortality for both mother and baby. Smoking is associated with low birth weight, miscarriage, placental abruption, pre-term birth, and neonatal morbidity and mortality. In addition, smoking during pregnancy is associated with long-term consequences for the child in terms of neurological development, endocrine dysfunction, and oncogenesis [1]. Babies born to smokers are more likely to suffer sudden infant death syndrome. Children of smokers have a higher incidence of childhood asthma, behavioural disorders, and poor academic performance in school [2, 3]. Children of smokers are also twice as likely to smoke themselves later in life [4].

For other risk-conferring antepartum medical conditions such as gestational diabetes mellitus (GDM), the standard of care is a dedicated antenatal clinic for treatment. This multidisciplinary team comprises an obstetrician, a diabetologist, midwife, diabetic nurse specialist, and dietician. A dedicated diabetic antenatal clinic has been shown to reduce the rate of serious perinatal complications for women with GDM [5]. Smoking is a risk-conferring antepartum condition strongly associated with complications such as fetal growth restriction. However, there is no international consensus on how to manage pregnancies complicated by smoking other than to offer smoking cessation support.

Many trials have studied smoking cessation in pregnancy, utilising methods such as psychological interventions, nicotine replacement therapy (NRT), group therapy, motivational interviewing, incentive-based therapy, feedback interventions, and exercise. These trials have been extensively summarised by Cochrane reviews [6, 7]. There are currently no international guidelines on ultrasound screening of smokers for fetal growth restriction during pregnancy, despite smokers having babies with a lower mean birth weight than non-smokers and a higher incidence of fetal growth restriction.

Behavioural interventions are more likely to succeed in an environment enhanced by supportive policies that contextualize the intervention—for example, interventions in smoke-free hospital campuses [8, 9]. We hypothesise that providing care to smokers in a dedicated smoking cessation antenatal clinic—‘the Smoking cessation Through Optimisation of clinical care in Pregnancy (STOP) clinic’—will result in a higher rate of smoking cessation compared to routine care. This higher rate of cessation will lead to increased birth weight and improved maternal and neonatal outcomes. This clinic will draw on the unique relationship enjoyed between a woman and her clinicians during pregnancy which provides for close patient contact over a period of time, creating a catalyst for change for many women. The clinic is the intervention, rather than a single specific psychological or pharmacological intervention.

## Methods

### Study design

This is an ongoing single-centre pragmatic randomised controlled trial (RCT) which commenced in February 2018. Written informed consent for each study participant is obtained prior to any data collection. The study is registered with the International Standard Randomised Controlled Trial Number clinical trial registry (ISRCTN 11214785). The Standard Protocol Items: Recommendations for Interventional Trials (SPIRIT) checklist is provided as Additional file 2.

### Pragmatic trial protocol

The STOP trial utilises a pragmatic RCT protocol. Pragmatic trials ascertain whether a difference exists in treatment as applied in clinical practice. These trials evaluate the beneficial effect of an intervention when applied by any clinician to any patient studied. The pragmatic features of the STOP RCT are as follows:

- Broad patient selection criteria, allowing enrolment of a heterogeneous patient population
- An intervention delivered by clinicians in a normal clinical setting, with a protocol that allows flexibility to adapt the intervention to individual patient needs, for example, the use of NRT by some patients
- The selection of a primary outcome that reflects a ‘real-world’ concern of both patients and clinicians: smoking cessation
- Non-blinding of clinicians involved in delivery of the intervention, as blinding is difficult in clinical practice due to differences in appearances of treatments, e.g. attending a specialised clinic versus attending a general clinic
- Analysis based on an intention-to-treat approach, which recognises that treatment crossovers occur in ‘real-world’ clinical practice.

### Population

This trial aims to recruit women who self-report smoking at least one cigarette per day and do not have any comorbidities requiring specialist antenatal care. The inclusion and exclusion criteria are detailed in Table 1.

**Table 1:** Eligibility criteria

Inclusion criteria:	
1	≥ 18 years old
2	Singleton pregnancy
3	Smoking ≥1 cigarette daily
4	English language spoken
Exclusion criteria:	
1	Significant maternal medical disorder, e.g. cardiac, haematological, or endocrine disease requiring specialised maternal antenatal care
2	Significant maternal psychiatric disorder, e.g. delusional or psychotic disorders, severe depression requiring hospitalisation, use of ≥ 2 psychotropic drugs for treatment
3	Serious comorbid addiction issues, e.g. opiate abuse, methadone maintenance program
4	Positive serology requiring specialised antenatal care
5	Significant fetal anomaly defined as aneuploidy, life-limiting, or lethal fetal anomaly
6	Intellectual disability or lack of capacity
7	Poor or no understanding of the English language

### Recruitment

Participants are recruited from the patient population at the Coombe Women & Infants University Hospital in Dublin, Ireland. We have multiple sources of recruitment, including:

- In-hospital midwife referrals at booking visit
- Community midwife booking referrals
- Referral from maternity wards of smoking patients admitted in early pregnancy
- Referral via ultrasonographers at first scan in the pregnancy
- Referral from consultant at first booking visit

### Informed consent

Pregnant smokers are identified at time of booking history and have routine ultrasound confirmation of an ongoing viable pregnancy. Once inclusion criteria and exclusion criteria are applied, the woman is met by a member of the research team and given verbal and written information on the study. If she agrees to take part, she signs a written consent form. Participants are free to leave the trial at any point, in which case they are transferred back to their referring clinician.

### Ethical approval

Ethical approval was granted by the Coombe Women & Infants University Hospital Research and Ethics Committee (Study No. 25-2017). All research is carried out in accordance with the Code of Ethics of the World Medical Association (Declaration of Helsinki).

### Sample size justification

Approximately 6% of women will achieve smoking cessation with individual behavioural support via a structured intervention during pregnancy [10]. Previous large-scale trials in the UK have used a figure of 9% cessation in pregnancy unaided, with a further 6% cessation arising from an intervention [11]. Longitudinal data on pregnancy in women who quit after the first antenatal visit and before late pregnancy gives a similar figure (14.6%) [10]. We aim to double the ‘routine care’ cessation rate of 9% to give an intervention cessation rate of 18%. In terms of creating a model of care for pregnant smokers, we feel it is important to have a substantial increase in cessation rates over routine care to make it a viable clinic. Assuming a 5% level of statistical significance and 80% statistical power, the sample size is 225 per study arm. In a sensitivity analysis, under the assumption of no dropouts, the trial would have higher statistical power of precisely 90% or alternatively allow detection of a lower cessation rate of 16% in those randomised to the smoking cessation clinic.

### Randomisation

**Fig. 1:**
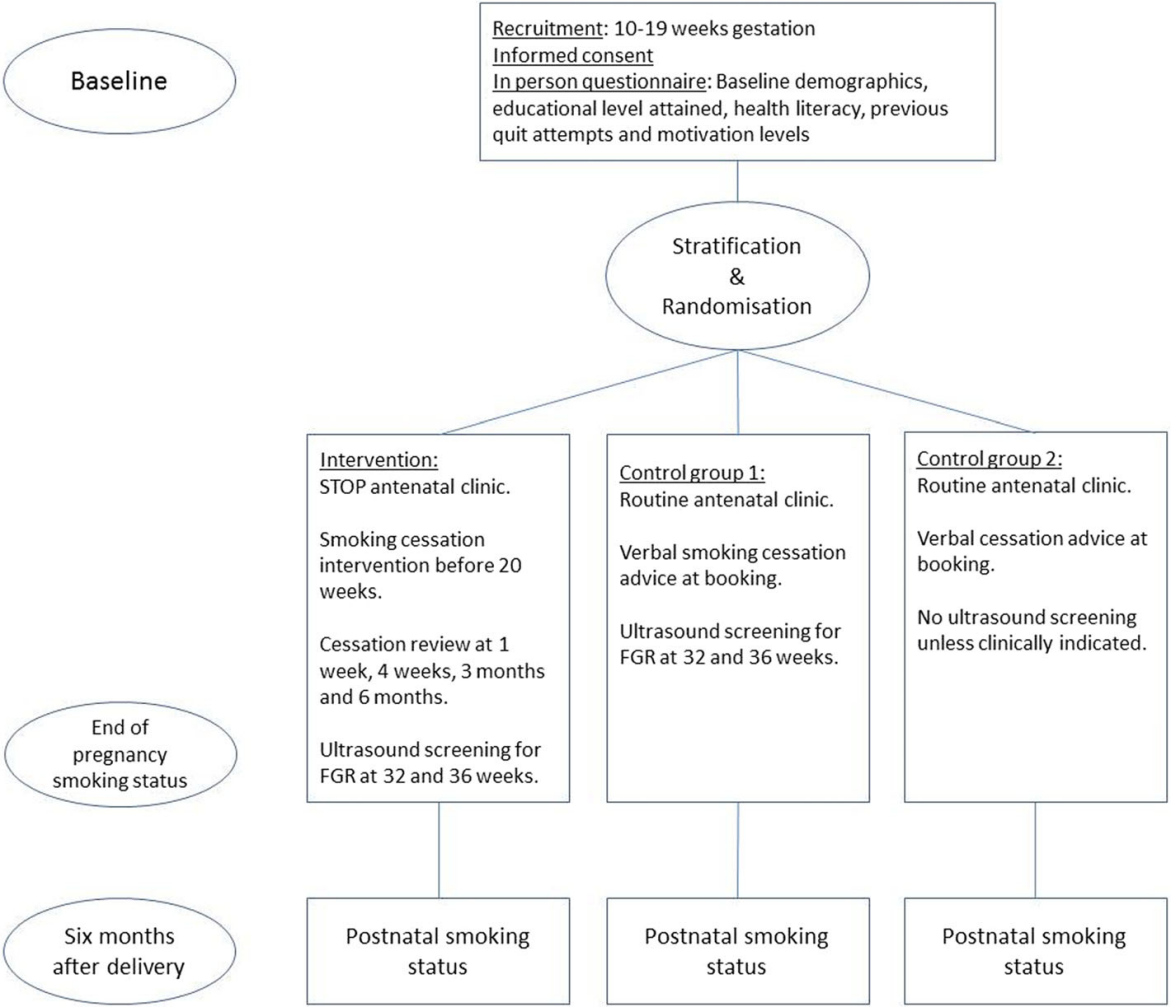
Trial schema

Patients are randomised on a secure data spreadsheet using a computer-generated random allocation list with allocation concealment. We are utilising block randomisation (block size of 4) with stratification for age, parity, and history of previous fetal growth restriction (see Fig. 1: trial schema).

### Blinding

The nature of the intervention does not permit blinding of either patient or clinician, but the data entry and statistical analysis are blinded procedures. This is achieved by data entry from an electronic database using study number only, rather than identifiable patient details. Additionally, the statistical analysis is carried out on a finalised database remote from the study centre and using study numbers only.

### Intervention

#### Study protocol

Patients in the STOP clinic have their booking visit between 10 and 19 weeks and see the obstetric team, midwife, and smoking cessation practitioner. Baseline demographics, educational level attained, health literacy, previous quit attempts, and motivation are assessed with a behavioural questionnaire. Data on the patient’s current smoking status and habits, including the use of other tobacco products and e-cigarettes, will be recorded and a measure of her level of addiction will be performed via the Fagerström test [12].

#### Clinic structure

At each visit the patient has her blood pressure measured and a dipstick urinalysis. Symphysio-fundal height (SFH) is recorded, liquor volume is assessed clinically, and the fetal heartbeat is checked. In cases of morbid obesity where the SFH is inaccurate, a single fetal abdominal circumference can be obtained. Fetal biometry will only be performed if clinically indicated—for example, if there is suspected growth restriction, macrosomia, or need for biometry secondary to history or additional risk factors. Patients will remain in the STOP clinic regardless of smoking status, quit attempts, or failure to quit.

**Fig. 2:**
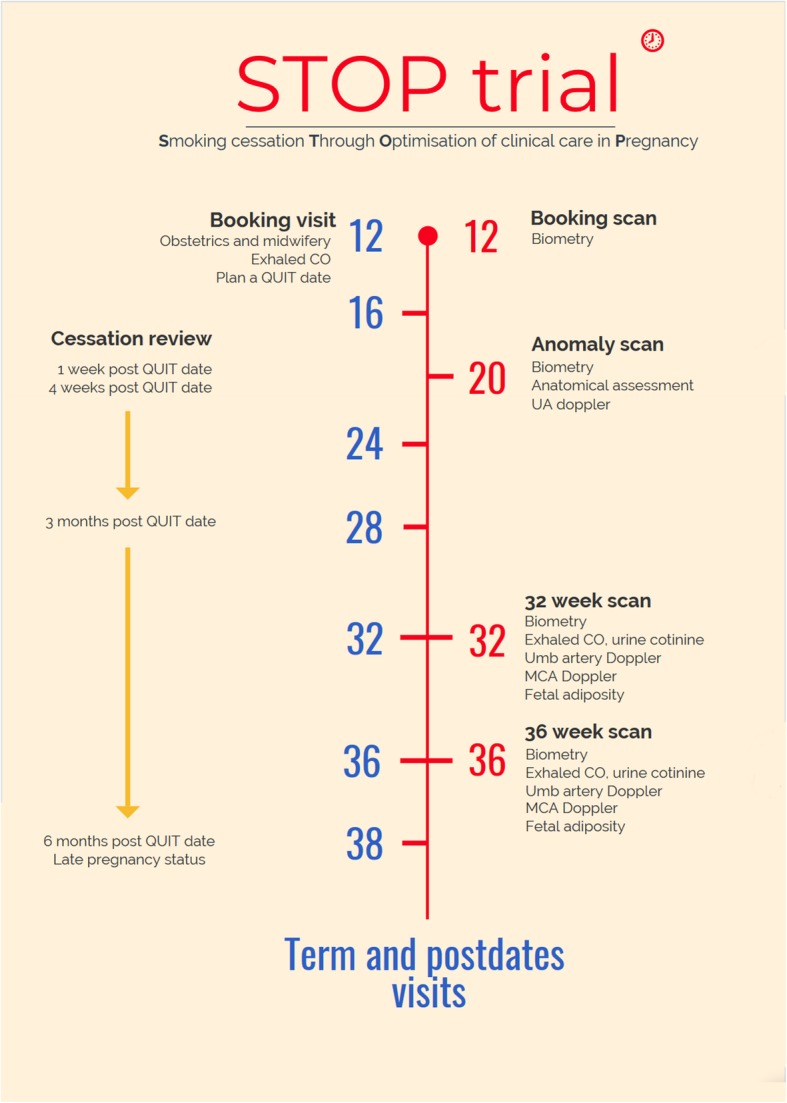
STOP trial clinic structure

**Fig. 3:**
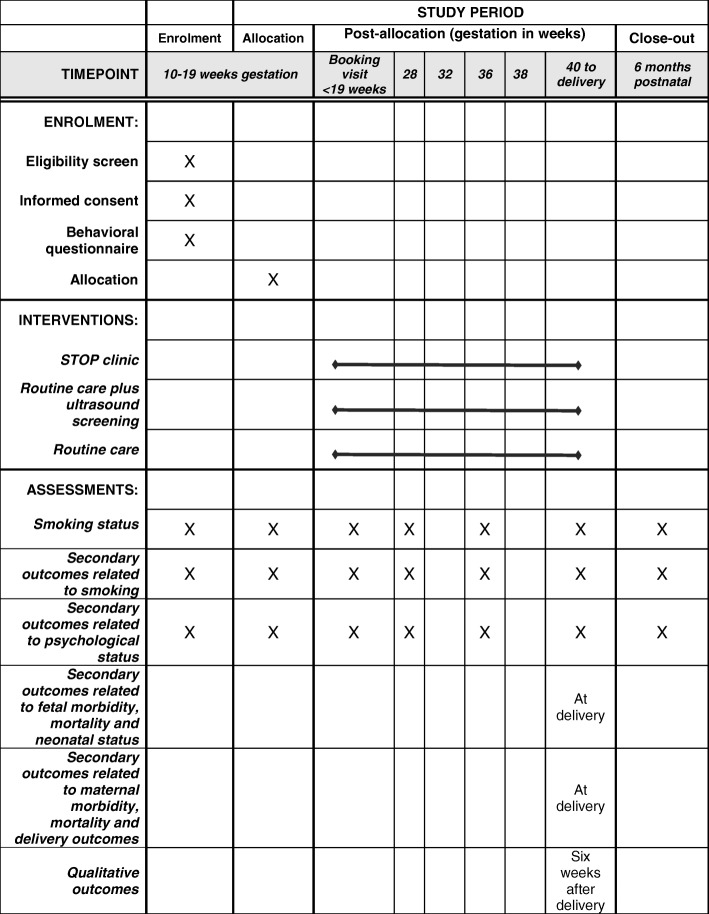
SPIRIT figure

Follow up antenatal visits are held at 28, 32, 36, 38, and 39–40 weeks and at postdates (Fig. 2). The schedule of assessments and interventions is shown in Fig. 3.

#### Ultrasound

An ultrasound scan is performed at booking (early second trimester) and between 20 and 22 weeks for anomaly screening. Additional growth scans are carried out at 32 and 36 weeks to screen for fetal growth restriction. Triplicate measures of biparietal diameter, head circumference, abdominal circumference, and femur length are obtained, and an estimate of fetal weight is calculated using the Hadlock formula [13]. Fetal adiposity is measured at the fetal thigh and abdomen as described previously [14]. Doppler measurements are obtained from both the umbilical and middle cerebral arteries. At the time of these growth scans, the self-reported number of cigarettes is recorded along with the timing of the last cigarette use. Exhaled carbon monoxide (CO) and urinary cotinine are measured. Images taken by the ultrasonographer are qualitatively controlled and scored according to the scheme described by Salomon et al. [15].

#### Smoking cessation program structure

A smoking cessation practitioner assesses the patient as part of the primary care giving team and measures exhaled CO. The Health Service Executive (HSE) Tobacco Cessation Support Program is a structured behavioural support program used by smoking cessation specialists in Ireland. It uses behavioural change techniques to support the tobacco user through the process of quitting by increasing confidence and motivation to quit and developing personal coping skills to sustain the quit attempt over time. Once the smoking cessation intervention is performed, a ‘quit date’ is set. The smoking cessation practitioner then sees the patient according to the following schedule:

- One week post quit date
- Four weeks post quit date
- Three months post quit date
- Six months post quit date/end of pregnancy (depending on gestation).

If a patient is still smoking at 4 weeks post quit date, NRT is offered in accordance with HSE guidelines which recommend its use in pregnancy only after a psychological intervention has failed.

The smoking cessation practitioner return visits are held in the clinic alongside the antenatal team. Current smoking status is recorded at each visit to the smoking cessation practitioner. If the patient has quit smoking, the timing of cessation and any aids used are recorded. Measures of urge to smoke, tobacco withdrawal score, and confidence in quitting are recorded. Quantitative measurement of urine cotinine is performed at 32 and 36 weeks, coinciding with the third trimester ultrasound scans. The HSE-adapted Russell Standard definitions of Quit are used [16]:

- *Self-reported Quit*. This is defined as a self-report of smoking not more than five cigarettes from the quit date. A standard abstinence question is ‘Have you smoked at all since (date of start of abstinence period) A: No, not a puff; B: 1–5 cigarettes; C: More than 5 cigarettes?’ Answer A or B can be classified as a Self-reported Quit.
- *Validated Quit*. A Self-reported Quit that is validated with a CO monitor reading of less than 10 ppm is classified as a Validated Quit.

### Midwifery

The STOP clinic delivers both obstetric and midwifery expertise. The midwife in the STOP clinic provides support during the pregnancy, information on labor and delivery, and breastfeeding information. The midwife also coordinates referrals to antenatal classes and liaises with the multidisciplinary team when required.

### Postnatal assessment and qualitative measures

After delivery, a member of the research team meets the patient to perform a CO test. A pseudonymised questionnaire is provided to record her current smoking habits, information on cessation (if it has occurred), and any aids used. Satisfaction with care, confidence in healthcare providers, and confidence as an active participant in healthcare decisions are recorded with Likert-type scales. An Edinburgh Postnatal Depression Scale score is also recorded.

A final cessation check is performed at 6 months postnatal to screen for recidivism and is validated by a CO measurement, in line with the Russell Standard for reporting in smoking cessation clinical trials [16].

### Controls

The control group is 226 patients booked and recruited at the same time and randomised to ‘routine care.’ These patients have smoking cessation advice given as a ‘once-off’ at the booking visit via verbal or written information on the HSE Quit service, which is the current routine care in the Coombe and all obstetric units in Ireland [17]. The patients will attend either a general obstetric clinic or midwifery-led clinic and have a booking scan and 20–22-week anomaly scan as normal. The control group will be divided into two groups: 113 will have an additional scan at 32 and 36 weeks to screen for intrauterine growth restriction (IUGR), and 113 will receive no additional scans unless clinically indicated by their team. This is done to ensure testing of the hypothesis that the clinic as the intervention is responsible for any difference in outcomes, rather than the provision of additional ultrasound scans.

### Data management

Data collected in the study will be irrevocably anonymised, with patient names, hospital numbers, and routine testing specimen numbers de-identified and replaced with study numbers for the purpose of this research. The collected data will be stored in a password-protected spreadsheet on an encrypted computer and will be exported as a dataset for statistical analysis.

### Data and safety monitoring

The STOP trial reports to a Data and Safety Monitoring Committee composed of two independent clinicians with no involvement in the study, an independent statistician and an independent psychologist.

## Analyses

### Statistical analysis

Outcomes in the smoking cessation intervention group will be compared to those for all control patients in an intention-to-treat analysis. A 5% level of significance will be used in a chi-square test for a difference in overall smoking cessation rates. Post hoc testing will consist of comparing the two control groups, i.e. controls under routine care versus controls with third trimester study scans (screening for IUGR). In a secondary analysis, IUGR rates will be compared between the smoking cessation group and the control group screened for IUGR. An analysis of covariance (ANCOVA) analysis will be used to adjust for the stratification factors (age, parity, and history of fetal growth restriction). Intervention adherence will be assessed, and the group comparisons will be reproduced in a per-protocol population. Potential patient selection bias will be assessed using the Berger-Exner test. Statistical analysis will be performed using the STATA IC15 statistical package.

### Primary outcome

The Russell Standard for reporting of smoking cessation in clinical trials will be followed [16].

The primary outcome is self-reported, continuous abstinence from smoking between the quit date and end of pregnancy, validated by exhaled CO or urinary cotinine. The quit date is targeted as being at or before 16 weeks’ gestation and no further than 28 weeks’ gestation.

Secondary outcomes are (see Additional file 1):

- *Smoking*. Number of cigarettes smoked. Smoking cessation at 3 months post quit date, 6 months post quit date and/or end of pregnancy. Smoking cessation at 6 months postpartum
- *Psychological*. Urge to smoke, tobacco withdrawal symptoms, self-confidence in stopping smoking, self-reported depression at end of pregnancy
- *Fetal morbidity and mortality including neonatal measures*. Miscarriage, stillbirth, neonatal death, birth weight, estimated fetal weight at 32 weeks, estimated fetal weight at 36 weeks, spontaneous pre-term birth, iatrogenic pre-term birth, birth injury, neonatal complication, oxygen dependence, admission to neonatal unit, length of stay of neonate. Modified from Core Outcome Measures in Effectiveness Trials (COMET) Initiative [18]
- *Maternal morbidity and mortality including delivery outcomes*. Maternal death, mode of delivery, need for induction/delivery, maternal need for intensive care, maternal length of stay, pre-eclampsia, pregnancy-induced hypertension, postpartum haemorrhage, blood transfusion, late maternal complication. Modified from COMET Initiative [18]
- *Qualitative measures*. Satisfaction with the results of care, confidence as an active participant in healthcare decisions, confidence in healthcare providers.

The secondary outcomes are defined in detail in Additional file 1.

## Conclusions

Maternal smoking rates are declining; however, smoking remains a significant risk factor for maternal and neonatal morbidity. A higher proportion of continued cigarette smoking is encountered in lower socioeconomic groups. Our previous research shows that the needs of pregnant smokers are largely unmet and under-resourced [19]. Smokers are a different population to non-smokers. They are more likely to be younger mothers, unemployed, with low educational attainment, a lack of social support, and increased incidence of mental illness [6]. Despite decades of research into smoking in pregnancy, the most effective antenatal model of care for smokers is still unclear. Additionally, while smokers are at significant risk of fetal growth restriction, specific recommendations for screening for growth restriction are absent.

Pregnant women are routinely offered specialised care and treatment for other risk-conferring conditions, for example, specialised clinics for diabetes, medical disorders, haematology, cardiology, and addiction. Our clinical trial aims to test a specific model of care for pregnant smokers, with smoking cessation as the primary outcome. We also expect that if a higher level of cessation is achieved, this will translate into improved secondary outcomes such as birth weight.

The main strength of this study is that it provides a smoking cessation intervention that is integral to the antenatal clinic model of care, meaning that women do not have to attend a separate appointment for smoking cessation advice. This should improve uptake of the smoking cessation intervention. There is a high rate of recidivism amongst quitters in pregnancy, with many women relapsing before the end of pregnancy and in the early postnatal period [20–22]. Less than a third of spontaneous quitters in pregnancy remain abstinent one year postpartum [22]. Women who are single and parous, who have a partner or household member who smokes, those with high depression scores, and those with a heavier smoking habit pre-pregnancy are most likely to relapse in the postpartum period [23, 24]. An additional strength of our trial is that the participants have a follow-up assessment of smoking status at 6 months after the birth.

An anticipated challenge for this study is retention of participants in the clinical trial, as our previous work has shown a relatively high rate of non-attendance amongst smokers for antenatal clinic appointments [19]. Additionally, should a smoker develop certain complications (most commonly, diagnosis of gestational diabetes requiring metformin or insulin treatment), she will require transfer out to another specialist antenatal clinic, leading to drop out.

This study may lead to an improved model of care for women who smoke in pregnancy as well as recognition that this risk factor requires specialised antenatal input from healthcare professionals.

### Trial status

The trial protocol is version 1, 01 February 2018. Recruitment began in February 2018, and the projected recruitment end date is April 2020.

## Additional files

Additional file 1: Secondary outcome definitions and details of measurement. (DOCX 17 kb)

Additional file 2: SPIRIT 2013 checklist: recommended items to address in a clinical trial protocol and related documents. (DOC 119 kb)

## Data Availability

The datasets generated and/or analysed during the current study are not publicly available due to limits set during the ethical review process but are available from the corresponding author on reasonable request.
