# Supplementary material for: Smoking cessation Through Optimisation of clinical care in Pregnancy: the STOP randomised controlled trial
Source: Trials. 2019 Sep 3;20:550. doi: 10.1186/s13063-019-3653-4 (PMC6724369; doi:10.1186/s13063-019-3653-4)
Supplement: Supplementary file 1 — Secondary outcome definitions and details of measurement. (DOCX 17 kb) [file 13063_2019_3653_MOESM1_ESM.docx]

**Additional file 1**

Secondary outcome definitions and details of measurement.

| **Smoking outcomes:** | | Measurement: |
| --- | --- | --- |
| 1 | Number of cigarettes smoked | Self-report at time of delivery. Exhaled carbon monoxide (CO) measured at same time. |
| 2 | Smoking cessation at 3 months post quit date | Self-report, validated by exhaled CO measured at same time. |
| 3 | 6 months post quit date and/or end of pregnancy | Self-report, validated by exhaled CO measured at same time. |
| 4 | Smoking cessation at 6 months postpartum | Self-report by telephone, with any quitters validated by exhaled CO measurement in person. |
| **Psychological:** | | |
| 1 | Urge to smoke | Assessed by questionnaire at first visit, 1 week, one month, 3 months and six months post quit date, if quit date set. |
| 2 | Tobacco withdrawal symptoms |  |
| 3 | Self-confidence in stopping smoking |  |
| 4 | Self-reported depression at end of pregnancy. | Assessed using Edinburgh Postnatal Depression score. |
| **Fetal morbidity and mortality including neonatal measures:** | | |
| 1  2  3  4 | Miscarriage  Stillbirth  Neonatal death  Birth weight | Data collected from electronic healthcare record after pregnancy completion, with reference to paper chart if required.  Miscarriage refers to pregnancy loss before 23+0 weeks gestation or with a birth weight less than 500g.  Stillbirth is defined an antepartum or intrapartum intrauterine death of an infant over 23+0 weeks gestation or with a birth weight greater than 500g.  Neonatal death is defined as the death of an infant after birth up to 28 days of life. |
| 5  6 | Estimated fetal weight at 32 weeks  Estimated fetal weight at 36 weeks | Data collected from Viewpoint™ (GE Healthcare).  Calculated using triplicate measures of biparietal diameter, head circumference, abdominal circumference, and femur length with an estimate of fetal weight calculated using the Hadlock formula as described in protocol. |
| 7  8 | Spontaneous pre-term birth  Iatrogenic pre-term birth | Pre-term birth is defined as birth before 37+0 weeks gestation. |
| 9 | Birth injury | Birth trauma – fractured bones, intracranial hemorrhage or extracranial injuries. |
| 10 | Neonatal complication | Meconium aspiration syndrome  Need for ventilated respiratory support  Neonatal infection  Neonatal seizures  Diagnosis of Hypoxic Ischaemic Encephalopathy and/or need for therapeutic hypothermia  Long-term disability including neurodevelopmental delay |
| 11 | Oxygen dependence | Dependence on supplemental oxygen at discharge of neonate |
| 12 | Admission to neonatal unit |  |
| **Maternal morbidity and mortality including delivery outcomes:** | | |
| 1 | Maternal death | Data collected from electronic healthcare record after pregnancy completion, with reference to paper chart if required.  Maternal death is defined as death of a woman while pregnant or within 42 days of end of pregnancy, irrespective of the duration and the site of the pregnancy, from any cause related to or aggravated by the pregnancy or its management, but not from accidental or incidental causes.  Definitions of pre-eclampsia and pregnancy induced hypertension as per NICE clinical guideline 133 (1).  PPH is defined as the loss of >500ml blood at time of delivery.  Late maternal complication is defined as new postpartum hypertension requiring treatment; infection requiring antibiotic therapy; retained products of conception; secondary PPH or need for postnatal readmission. |
| 2 | Mode of delivery |  |
| 3 | Need for induction/delivery |  |
| 4 | Maternal need for intensive care |  |
| 5 | Maternal length of stay |  |
| 6 | Pre-eclampsia |  |
| 7 | Pregnancy induced hypertension |  |
| 8 | Postpartum hemorrhage |  |
| 9 | Blood transfusion |  |
| 10 | Late maternal complication |  |
| **Qualitative measures:** | | |
| 1 | Satisfaction with the results of care | Assessed by Likert-scale questionnaire sent by post after discharge of patient. |
| 2 | Confidence as an active participant in healthcare decisions |  |
| 3 | Confidence in healthcare providers |  |

1. National Institute of Clinical Excellence. Hypertension in pregnancy: diagnosis and management. Nice Guideline 107. Published August 2010. Available from https://www.nice.org.uk/guidance/cg107.
